# Trends of incident stimulant use disorder diagnoses before and after the COVID-19 pandemic in British Columbia (2013-2024): a population-based study

**DOI:** 10.3389/fpsyt.2026.1682481

**Published:** 2026-05-07

**Authors:** Roshni Desai, Kevin Hu, Bin Zhao, Shania Au, Xuan Chen, Mallory Flynn, Mike Irvine, Hind Sbihi, Brooke Kinniburgh, Kali-Olt Sedgemore, Kirsten Marchand, Sofia Bartlett, Naveed Janjua, Alexis Crabtree, Heather Palis

**Affiliations:** 1 British Columbia (BC) Centre for Disease Control, Vancouver, BC, Canada; 2 School of Population and Public Health, University of British Columbia, Vancouver, BC, Canada; 3 Foundry, Vancouver, BC, Canada

**Keywords:** cocaine, COVID-19, interrupted time series, methamphetamines, pandemic, stimulant use, stimulant use disorder, amphetamines

## Abstract

**Background:**

There is rising detection of unregulated stimulants (e.g. cocaine and methamphetamine) in toxicology results among people who died of unregulated drug poisoning. Nevertheless, little research describes the population-level trends of incident (new) stimulant use disorder (StUD) diagnoses. This study reports on trends of incident StUD diagnoses pre- and post-Covid-19 public health emergency in British Columbia (BC), Canada.

**Methods:**

Interrupted time series analyses were conducted with BC’s COVID-19 public health emergency declaration on March 16, 2020 as the interruption point. Descriptive statistics on demographic and health service contact were conducted for the population diagnosed before (January 1, 2013 - March 16, 2020) and after (March 17, 2020 - December 31, 2024) the COVID-19 pandemic emergency declaration. Seasonal autoregressive integrated moving average (sARIMA) models were used to .estimate changes to incident StuD diagnoses rates before and after the COVID-19 pandemic declaration.

**Results:**

38, 217 people were identified with incident StUD diagnoses between January 1, 2013 and March 31, 2024. The average diagnosis rate of incident StUD was 5.18 per 100, 000 in the pre-pandemic period and increased by 19.9% to 6.21 per 100, 000 in the post-pandemic period. The estimated increase in slope (ramp) of incident StUD was 0.0315 cases per 100, 000 population per month (95% CI: -0.00182, 0.06482).

**Conclusions:**

We identified a rate of increase in incident StUD diagnoses since the COVID-19 pandemic declaration in BC that was not statistically significant. Our study highlights the need for more comprehensive linked data -including, administrative health data, surveys, and other services/program data (e.g., community services, private sector) to better disentangle StUD incidence and prevalence to inform services to meet the needs of people with StUD. Stimulant use, Stimulant use disorder, pandemic, Covid-19, methamphetamines, cocaine, interrupted time series.

## Introduction

Evidence suggests rising harms due to unregulated stimulant use at a population-level, including increasing proportions of methamphetamines detected in toxicology among people who died of unregulated drug poisoning in British Columbia (BC), Canada. According to recent reports from the BC Coroners Service, the percentage of all unregulated drug deaths where methamphetamine was detected increased from 31% in 2017 to 50% in 2024. Cocaine, while initially decreasing between 2016 – 2022 (47% to 40%), has also increased in the last two years (45% in 2024) (1).

While post-mortem toxicology provides information about unregulated stimulant harms, there is less information available on population level rates of stimulant use or rates of stimulant use disorder (StUD) diagnoses, particularly in the context of the COVID-19 pandemic. The National Survey on Drug Use and Health (NSDUH), based in the United States reported overall increases in ‘illicit drug use’ among people aged 12 and older since the pandemic (2). Other survey-based research studies have reported changes in the prevalence or patterns of unregulated substance use during the pandemic. These studies appear to have mixed results, with some reporting increases in the frequency of substance use due to isolation and others reporting reductions (3–6). Studies on changes to reported rates of stimulant use since the pandemic are similarly mixed (5–8). The reasons behind these differing results may include region, setting (rural vs urban), context of use (e.g. regularly using vs using only in recreational settings), specific substance (decreases in cocaine compared to increases in methamphetamine use) or the population being studied (5, 9, 10) given motivations for stimulant use are known to vary across populations (11).

Additionally, limited research has characterized population-level trends in incident stimulant use disorder (StUD), including the demographic, chronic disease, and mental health service utilization profiles of people newly diagnosed with this condition. Acute and chronic mental and physical health conditions have been associated with stimulant use, and it is therefore critical to examine these conditions to make sense of any potential changes in the profile of people using stimulants and diagnosed with StUD in BC (12). Furthermore, the COVID-19 pandemic was known to pose interruptions to care, including care for people with substance-related healthcare risks (13). Pandemic related closures disrupted the illicit drug market, potentially affecting availability and quality of substances and subsequently impacting use rates and harms resulting from the unregulated drug supply (13). We therefore sought to examine the physical and mental health service contacts of people with incident StUD diagnoses in the pre- and post-pandemic periods, in both hospital and primary care settings, to identify potential changes to the profile of patients diagnosed in recent years, to inform an understanding of their substance-related healthcare needs.

This study reports on trends of incident StUD diagnosis (herein referred to as StUD diagnosis) pre- and post-pandemic in British Columbia (BC), Canada, and reports on the demographic and health profile of people. We hypothesize that the level and slope of stimulant use disorder rates will increase in the post pandemic period as other indicators of stimulant use such as stimulants deemed relevant to unregulated drug deaths have increased during this period.

## Methods

### Study sample

We used data from the Provincial Health Services Authority (PHSA) PANDA platform which integrates several individual-level anonymized administrative health care records including those related to hospitalization, outpatient billing, emergency department visits, pharmacy dispensations and demographic information such as birth and death records. PANDA evolved from the BC COVID-19 Proof of Concept and leverages BC’s universal health care system integrating administrative data on hospitalizations, outpatient visits, emergency health care and other health care records for all residents throughout the province (14).

### Stimulant use disorder diagnosis

The cohort of people with stimulant use disorder was derived via ICD 9 & ICD-10-CA codes in the Discharge Abstract Database (DAD, hospitalization) or Medical Services Plan (MSP, outpatient billing) where the diagnosis date was between January 1, 2013 and March 31, 2024 and the person was aged 12 or older on the record date. Diagnosis was determined where there was at least one hospitalization (DAD) or two or more outpatient visits (MSP record) within a year with the relevant ICD 9 or ICD-10-CA codes. The earliest record with codes for stimulant use disorder was used as the ‘diagnosis date’ (See **Table 1** for full list of codes). Demographic information was extracted from the BC Client Roster, which collects sociodemographic information for people registered with MSP. Health care utilization in the five years prior to diagnosis date was retrieved from outpatient (MSP) or hospitalization (DAD) records with ICD codes for each mental health or chronic condition Population estimates to derive the incidence rates were retrieved from BC Stats Population Estimates (See **Supplementary Tables S1–S4**).

**Table 1:** ICD-10-CA and ICD-9 codes for identification of stimulant use disorder.

	Hospitalizations, (ICD-10-CA)	Outpatient care (ICD-9)
Mental and Behavioural disorders due to use of cocaine Mental and Behavioural disorders due to use of other stimulants including caffeine	F14 (0-9) F15 (0-9)	
Drug Dependance: Cocaine Drug Dependance: Amphetamine type and other Psychostimulants Non-dependent [use] of drugs: Cocaine Type Non-dependent [use] of drugs: Amphetamine Type		304.2 304.4 305.6 305.7

A person was defined as a StuD case if they had two outpatient records within a year, in MSP or 1 hospitalization record in DAD with ICD-9 codes (for MSP) or ICD-10-CA codes (DAD) as listed in the table below.

### Statistical analysis

Individual characteristics pre- and post- pandemic were compared using Chi- Square tests for association. People diagnosed with StuD prior to the COVID-19 pandemic public health emergency declaration on March 16, 2020 in BC were in the pre-pandemic group and people diagnosed on or after this date were in the post pandemic group. Average monthly diagnosis rates in the pre- and post-pandemic periods were calculated and percentage change between the periods were compared by demographic characteristics.

**Figure 1:**
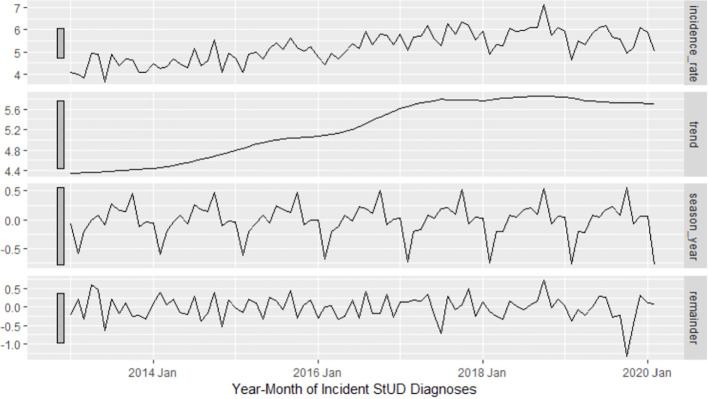
Seasonal-trend decomposition using Loess (STL) plot for StuD time series (pre-pandemic).

For the Interrupted Time Series analysis (ITS), the date of the COVID-19 public health emergency declaration (March 16, 2020) was used as the start of the interruption point. Monthly StUD diagnosis rates as defined above were used as the outcome. We first identified the model that best fit the data prior to the pandemic (January 2010-February 2020). The time series was decomposed using seasonal-trend decomposition using Loess (STL) on the monthly StUD incidence to examine potential trending and seasonality (**Figure 1**). The STL identified a clear upward trend in the data, which appeared stationary after differencing, and seasonality. We plotted the autocorrelation and partial autocorrelation functions on the remainder component identified by STL.

We estimated changes in the trend of StUD after the interruption using a seasonal autoregressive integrated moving average (sARIMA) model as autocorrelation was present in the time series. sARIMA captures the influence of previous values through its autoregressive (AR) component and accounts for the impact of past random errors with the moving average (MA) component. It extends this by incorporating seasonal AR and MA terms to model repeating seasonal patterns. Additionally, the differencing parameters help handle underlying trends and seasonality in the time series, making the series more stationary (15). We considered a model that was determined by examining the autocorrelation (ACF) and partial autocorrelation functions (PACF) of the remainder components from STL and a model chosen by the autoselection feature from the “forecast” package in R (see **Supplementary Figure S1**). Autoselection is based on the Hyndman-Khandakar algorithm, and selects the best model based on corrected AIC (AICc) score (16–18). The model that best fit the pre-pandemic data (January 2010-February 2020) represents the counterfactual trend had the interruption not occurred. This model was then applied to the full time series (January 2010-March 2024). Step (immediate changes in the month following the interruption, that then sustain at the same level) and ramp (changes to slope, where the time series changes by a certain amount per month) effects were then assessed and included based on AIC score using the same sARIMA model. All analyses were conducted using R 4.3.1 and the “fable” package was used for the ITS (18, 19).

## Results

A total of 38,217 people were newly diagnosed with StUD between January 1, 2013 and March 31, 2024. Of these, 42.4% were diagnosed in the 4 years since the pandemic was declared compared to 57.6% who were diagnosed in the 7 years prior to the pandemic declaration. Most people identified with StUD were male (66.5%) and aged between 25 and 54 (71.8%) at time of diagnosis (**Table 2**). A slightly higher proportion of people diagnosed in the post pandemic period were male (67.3% post vs 65.9% pre, p = 0.003). Youth (ages 12–18 and 19-24) represented a lower percentage of the people diagnosed in the post pandemic period (12.9% post vs 19.3% pre, p <0.001).

**Table 2:** Characteristics of people newly diagnosed with stimulant use disorder (StUD) by time period.

	Pre - pandemic ^4^, N = 21,998^1^ (57.6%)	Post - pandemic ^4^, N = 16,219^1^ (42.4%)	Overall, N = 38,217^1^ (100%)	p-value^2^
Demographics				
**Sex^6^**				0.003
Female	7,477 (34.0%)	5,286 (32.6%)	12,763 (33.4%)	
Male	14,501 (65.9%)	10,922 (67.3%)	25,423 (66.5%)	
**Age Group** ^5^				<0.001
12-18	966 (4.4%)	465 (2.9%)	1,431 (3.7%)	
19-24	3,279 (14.9%)	1,629 (10.0%)	4,908 (12.8%)	
25-34	6,799 (30.9%)	4,954 (30.5%)	11,753 (30.8%)	
35-44	5,024 (22.8%)	4,285 (26.4%)	9,309 (24.4%)	
45-54	3,684 (16.7%)	2,700 (16.6%)	6,384 (16.7%)	
55+	2,246 (10.2%)	2,186 (13.5%)	4,432 (11.6%)	
**Health Authority** ^5^				<0.001
Interior	3,575 (16.3%)	2,759 (17.0%)	6,334 (16.6%)	
Fraser	5,492 (25.0%)	3,922 (24.2%)	9,414 (24.6%)	
Vancouver Coastal	5,157 (23.4%)	3,098 (19.1%)	8,255 (21.6%)	
Island	2,465 (11.2%)	2,373 (14.6%)	4,838 (12.7%)	
Northern	1,598 (7.3%)	1,169 (7.2%)	2,767 (7.2%)	
Out of Province	718 (3.3%)	488 (3.0%)	1,206 (3.2%)	
Unknown	2,993 (13.6%)	2,410 (14.9%)	5,403 (14.1%)	
Health Service visits in the 5 years prior to StUD diagnosis				
Physician Visits for Mental Health Conditions ^3^				
**ADHD**	1,602 (7.3%)	1,659 (10.2%)	3,261 (8.5%)	<0.001
**Depression**	11,377 (51.7%)	7,703 (47.5%)	19,080 (49.9%)	<0.001
**Mood & Anxiety**	11,830 (53.8%)	8,361 (51.6%)	20,191 (52.8%)	<0.001
**Opioid Use Disorder**	4,080 (18.5%)	3,742 (23.1%)	7,822 (20.5%)	<0.001
**Substance Use Disorder**	8,719 (39.6%)	5,611 (34.6%)	14,330 (37.5%)	<0.001
**Schizophrenia**	3,591 (16.3%)	2,350 (14.5%)	5,941 (15.5%)	<0.001
Physician Visits for Chronic Conditions ^3^				
**Osteoarthritis**	1,584 (7.2%)	914 (5.6%)	2,498 (6.5%)	<0.001
**Rheumatoid Arthritis**	439 (2.0%)	231 (1.4%)	670 (1.8%)	<0.001
**Asthma**	2,566 (11.7%)	1,513 (9.3%)	4,079 (10.7%)	<0.001
COPD	1,440 (6.5%)	965 (5.9%)	2,405 (6.3%)	0.018
**Diabetes**	1,248 (5.7%)	913 (5.6%)	2,161 (5.7%)	0.9
**Heart Disease**	1,562 (7.1%)	1,188 (7.3%)	2,750 (7.2%)	0.4
**Heart Failure**	573 (2.6%)	536 (3.3%)	1,109 (2.9%)	<0.001
**Hypertension**	2,009 (9.1%)	1,621 (10.0%)	3,630 (9.5%)	0.005
**Kidney Disease**	931 (4.2%)	897 (5.5%)	1,828 (4.8%)	<0.001
Hospitalizations for Mental Health Conditions ^3^				
**ADHD**	216 (1.0%)	150 (0.9%)	366 (1.0%)	0.6
**Depression**	1,176 (5.3%)	672 (4.1%)	1,848 (4.8%)	<0.001
**Mood & Anxiety**	2,025 (9.2%)	1,278 (7.9%)	3,303 (8.6%)	<0.001
**Opioid Use Disorder**	430 (2.0%)	394 (2.4%)	824 (2.2%)	0.002
**Substance Use Disorder**	307 (1.4%)	423 (2.6%)	730 (1.9%)	<0.001
**Schizophrenia**	447 (2.0%)	286 (1.8%)	733 (1.9%)	0.058
Hospitalizations for Chronic Conditions ^3^				
**Osteoarthritis**	154 (0.7%)	99 (0.6%)	253 (0.7%)	0.3
**Asthma**	190 (0.9%)	63 (0.4%)	253 (0.7%)	<0.001
**COPD**	211 (1.0%)	143 (0.9%)	354 (0.9%)	0.4
**Diabetes**	182 (0.8%)	167 (1.0%)	349 (0.9%)	0.040
**Heart Disease**	173 (0.8%)	148 (0.9%)	321 (0.8%)	0.2
**Heart Failure**	142 (0.6%)	131 (0.8%)	273 (0.7%)	0.063
**Hypertension**	379 (1.7%)	231 (1.4%)	610 (1.6%)	0.021
**Kidney Disease**	61 (0.3%)	62 (0.4%)	123 (0.3%)	0.073
**Hospitalized Stroke**	55 (0.3%)	33 (0.2%)	88 (0.2%)	0.3

^1^n (%).

^2^Fisher's Exact Test for Count Data with simulated p-values (based on 2000 replicates); Pearson's Chi-squared test

^3^Excludes visits on same day as StUD Dx

^4^Pre - pandemic dates= January 1, 2013 – March 16, 2020

^4^Post - pandemic dates= March 17, 2020 – March 31, 2024

^5^ Age and Health Authority of Residence at time of Diagnosis via Client Roster

^6^ 29 people had unknown sex and excluded from this table

When considering physician visits, people diagnosed in the post-pandemic vs. pre-pandemic period were significantly less likely to have had a visit for all the mental health conditions examined, apart from ADHD. ADHD related visits were more prevalent among people diagnosed in the post-pandemic period (10.2% post vs 7.3% pre, p<0.001). Visits for all chronic health conditions were lower in the post-pandemic period, except for heart failure (3.3% post vs 2.5% pre, p <0.001), hypertension (10.0% post vs 9.1% pre, p=0.005), and kidney disease (5.5% post vs 4.2% pre, p <0.001).

The proportion of people with a history of hospitalizations for various mental health and chronic conditions was low overall and across both groups. When comparing people with StUD diagnoses in the post vs pre-pandemic period, the proportion with a history of hospitalizations was similar for all mental and chronic conditions examined (**Table 2**).

**Figure 2:**
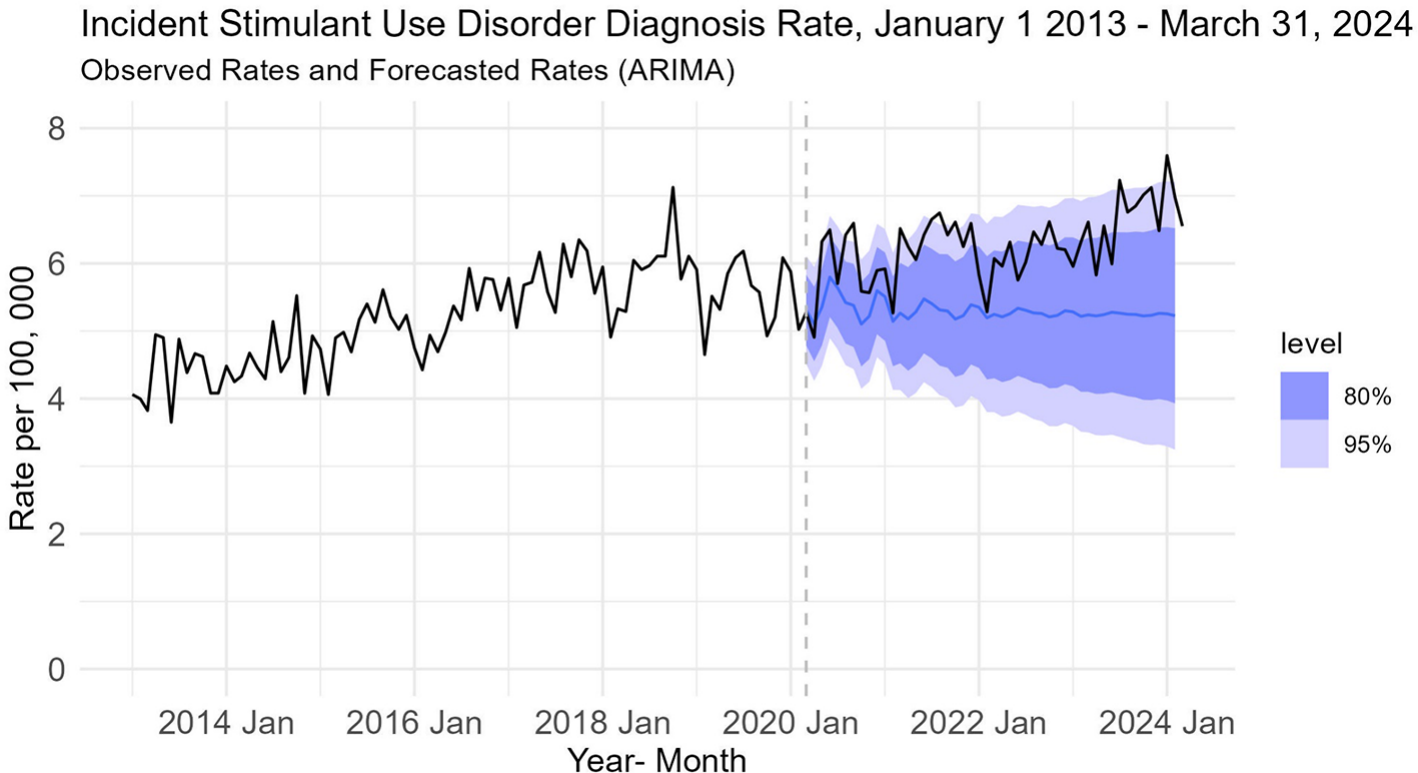
Observed and forecasted incidence rates of StUD forecasted rates (purple line) and 95% prediction interval (purple) are based on autoregressive integrated moving average (ARIMA) model fitted to the observed rates before March 2020 (represented by the dashed grey line). The pre pandemic model was then applied to the full pre-pandemic + post-pandemic data to forecast rates as shown by the purple line. The observed rates (shown by the black line), while trending upwards were all within in the 95% prediction interval of the forecasted rates.

Descriptive charts for StUD depicted a slow generally upward trend in the monthly diagnosis rate over the study period. Monthly rates increased between 2013–2018 and 2021-March 2024, but there was a period of stability between 2018-2021. The average diagnosis rate of StuD was 5.18 per 100, 000 population in the pre-pandemic period and increased by 19.9% to 6.21 per 100, 000 in the post-pandemic period (**Figure 2**).

### STL & interrupted time series analysis

The trend component of the STL showed small steady increases in the number of incident cases per 100, 000 population per month (4.4 per 100, 000 cases per month in January 2013 to 5.6 per 100, 000 cases per month in January 2017) which then appeared to flatten until the end of the study period (March 2024). The seasonal component identified some seasonal variation with the highest incidence rate occuring in the fall months, followed by decreases in winter months. No pattern was observed was observed in the remainder component (**Figure 1**).

Based on AICc scores, the model that best fit the pre-pandemic model was the one selected by the autoselection feature. To estimate potential ramp and step effects, we applied the sARIMA model that best fit the pre-Covid 19 data to the full range of data pre and post COVID. The model with only ramp had the lowest AICc (as well as AIC and BIC) scores compared to models with a step only or with both step and ramp. Based on this model, the estimated slope increase of StUD was 0.0315 cases per 100, 000 population per month (95% CI: -0.00182, 0.06482) post declaration of the COVID-19 pandemic. This was not a statistically significant change (**Figure 2**).

### Changes in rates by demographics

The new diagnosis rates for males were approximately two times higher among males (pre: 6.89 per 100, 000; post: 8.46 per 100, 000) compared to females (pre: 3.49 per 100, 000; post: 4.01 per 100, 000) in both the pre- and post-pandemic periods. Average monthly rates increased from the pre- to post-pandemic periods for both males and females, though the rate of change was higher for males (22.8%) compared to females (14.8%) (See **Table 3** and plot in **Supplementary Figure 2**).

**Table 3:** Average StUD incidence rates by demographic and time period.

Demographic	Pre-pandemic (per 100, 000)	Post-pandemic (per 100, 000)	Percent Change
Sex			
Female	3.49	4.01	14.8%
Male	6.89	8.46	22.7%
Age Group			
12-18	3.11	2.42	-22.4%
19-24	9.77	8.04	-17.7%
25-34	11.38	13.43	18.0%
35-44	9.05	11.98	32.4%
45-54	6.04	8.27	36.9%
55+	1.65	2.50	51.0%
Health Authority			
Interior	5.31	6.66	25.4%
Fraser	3.49	3.96	13.5%
Vancouver Coastal	5.00	4.99	-0.0%
Island	3.48	5.45	56.8%
Northern	6.25	7.87	25.9%

**Figure 3:**
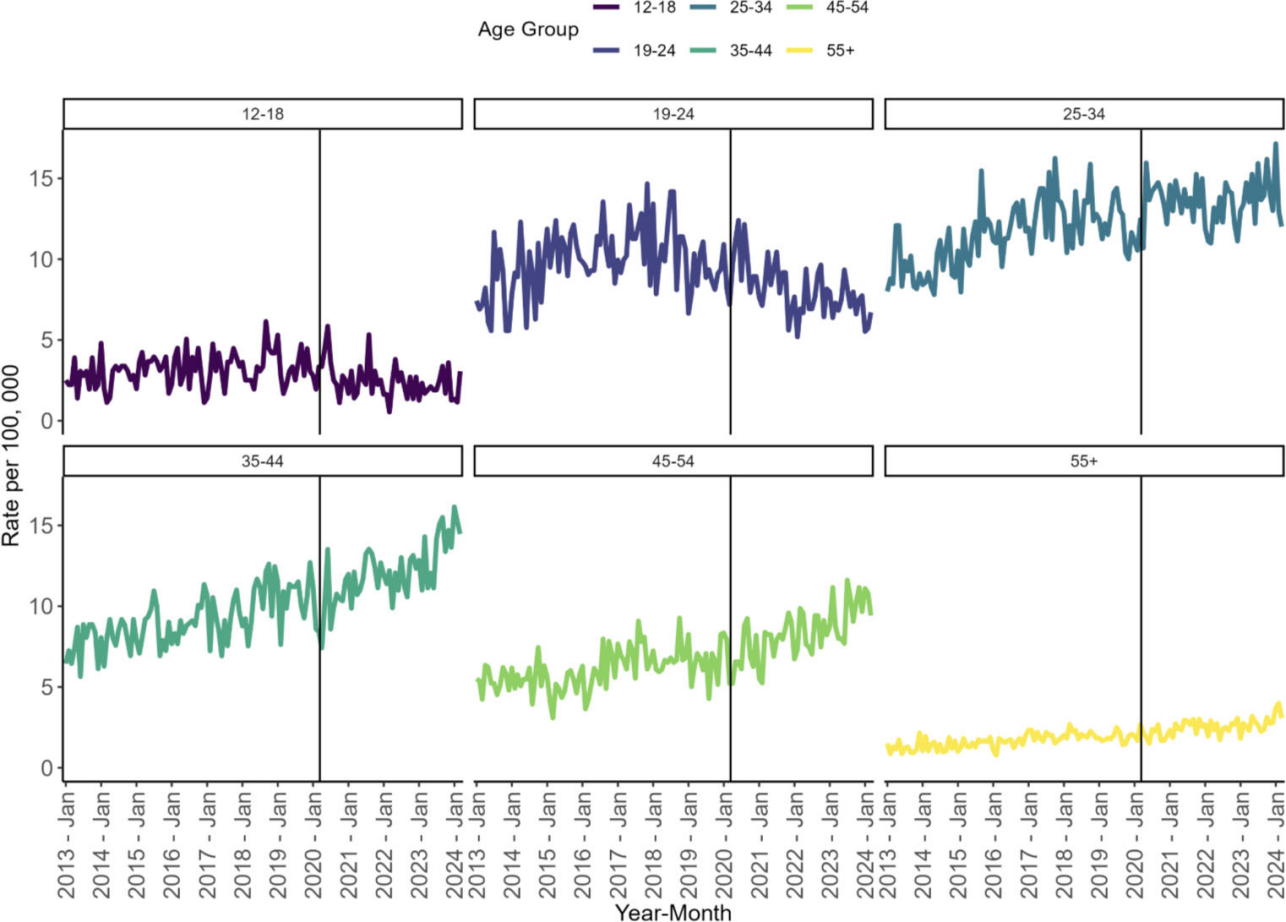
Observed incidence rates of StUD by age group.

In the post pandemic period the highest average new StUD diagnosis rates were observed in the 25-34- (8.04 per 100, 000) and 34-44- (13.43 per 100, 000) year age groups. Monthly rates increased across all age groups, except for youth (12–24) where the average monthly rate decreased in the post pandemic period (See **Table 3**; **Figure 3**).

## Discussion

In this study, we sought to identify people diagnosed with stimulant use disorder, health characteristics of people identified by our algorithm and changes in trends since the Covid-19 pandemic declaration in BC. While BC Coroner indicate that unregulated drug deaths with stimulants deemed relevant to the death have increased since the pandemic, the reasons for this increase are unknown. Qualitative research has found that among people who also use opioids, people could also be using stimulants for safety reasons, to improve energy levels, or given the false perception that stimulants might mitigate opioid drug poisoning risk (20, 21). Community reports also point to cross-contamination i.e. where the person intended to use stimulants but due to the unregulated drug supply or sharing of paraphernalia, consumed stimulants contaminated with fentanyl, although drug checking data has not demonstrated a parallel increase in expected stimulants testing positive for fentanyl during the study period (22).

The COVID-19 pandemic public health emergency declared in BC in March of 2020 resulted in public health restrictions intended to minimize exposure to the virus in communities (13). During the same period, an increase in the toxicity of the drug supply was observed, along with increases in unregulated drug poisoning events and deaths (13, 23, 24). Public health measures may have contributed to loss of employment, isolation, depression, and/or boredom which are commonly associated with substance use (13, 25–27).

We found that the incident StUD diagnosis rate was slowly increasing in BC before the COVID-19 pandemic and that the rate of the increase post-pandemic was not statistically different compared to the pre-pandemic period (ramp: 0.0315, 95% CI: [-0.00182, 0 0.06482]). We expected to see increases in StUD diagnoses due to the higher proportions of unregulated deaths with stimulants deemed relevant to death in post pandemic. However, in our study the rate of change of StuD diagnoses post pandemic was not statistically significant. There are several plausible reasons for this. Prior studies describe most substance use as recreational, with a low proportion of people reporting substance use developing a substance use disorder (28, 29). Furthermore, diagnoses are known to lag, and it is possible that increases in use will be reflected as new diagnoses in future years (30, 31). The relatively minor change in incident StUD trends may reflect limited services available for StUD compared to other SUDs, leaving people with StUD undiagnosed, given our definition depends on having health service records with a diagnostic code for StUD. For example, data from the OUD cascade of care report that 71% of people diagnosed with OUD received opioid agonist therapy (32), and in prior analyses we identified 74.5% of people with OUD were first identified via OAT dispensation records (33). In contrast, while no pharmacotherapies have been licensed for the treatment of StUD in North America, guidance on prescribing these medications off-label to people with StUD has been provided by the American Academy of Addiction Psychiatry and BC Centre on Substance Use in BC, with careful consideration of the potential risks and benefits. Nevertheless, few such options for StUD have been implemented in BC (34–36).

In terms of demographics, a significantly higher proportion of people diagnosed with StUD post declaration of the COVID-19 pandemic were in the older age categories. Diagnosis rates increased in the post-pandemic period among all age groups, except for youth (aged 12–24 years). In our study, youth rates decreased by 22.4% and 17.7% among youth 12-18- and 19–24-year-olds respectively. In general, this finding is consistent with a systematic review, which concluded that the prevalence of substance use among youth declined during the pandemic as well as findings from the National Survey on Drug Use and Health, which found decreases in past year use of cocaine (0.42% in 2018/19 to 0.14% in 2021/22) and methamphetamine (0.17% in 2018/19 to 0.10% in 2021/22) among youth post pandemic (2, 37). However, this trend has not been consistent across studies, likely due to meaningful differences in study samples, measures of substance use, and study time periods. This lack of consistent findings makes it difficult to draw firm conclusions on how the pandemic truly impacted substance use patterns and harms among youth This point is particularly important given that longitudinal studies have observed adverse impacts of the pandemic on youth with higher substance use risk profiles (e.g., those with concurrent mental health conditions) (38).

Considering these nuances, it is possible that the results observed among youth in our study are due to the hypotheses outlined above relating to barriers to service access (39). Prior research has shown that youth encounter significant barriers to accessing evidence-based harm reduction services and treatment for substance use disorders compared to adults (40–42). Commonly reported barriers, such as limited youth-specific services and specialist availability (e.g., pediatric addiction medicine, pediatric psychiatry, etc.), may have been particularly salient during the pandemic. The patterns observed among youth in our study may also be explained by shifts in service delivery settings for youth mental health and substance use. Recent administrative health data in Canada have shown that ED visits and hospitalizations for mental and substance disorders decreased by 31% and 23%, respectively, in 2023–2024 compared to 2018-2019, while outpatient physician visits increased by 8% (39). This shift may have led to lower diagnosis rates of StUDs as emerging evidence suggests that primary care providers face challenges with substance use screening/assessment, such as insufficient time, resources, training, and skills (43–45). The vast majority of people with substance use disorders, including StUD, do not access care for their StUD, and those who do, often do not receive evidence-based care (46, 47).

When considering health service contacts, we found that hospitalizations for all cardiac-related conditions were lower among people diagnosed in the post-pandemic period, while physician visits for hypertension and heart failure appeared to be higher. The higher proportion of physician visits for hypertension appears to contradict other studies which showed stable or lower physician visits for various conditions, including cardiac conditions among the general population post-pandemic (48, 49). Hypertension has been demonstrated to be elevated among people using stimulants thus this finding may suggest an increased cardiac service need among this population in the post pandemic period (50), particularly where cardiac co-morbidities may be exacerbated by or contribute to more severe COVID-19 (51).

Similarly, outpatient visits for ADHD were higher among people diagnosed post-pandemic declaration. This finding is in line with other studies that have shown increases in ADHD-related health care access and diagnosis since the pandemic was declared (52). Although history of SUD outpatient visits was lower among the post-pandemic group, SUD hospitalizations were higher compared to the pre–pandemic group. This may be related to the increase in drug poisonings that occurred during the pandemic which have been attributed to a number of factors, including increasing toxicity of the unregulated drug supply (1, 13, 53). This reflects the need to consider the syndemic effects for other public health emergencies (i.e. drug toxicity crisis) during pandemics (53).

### Limitations

This study has a number of important limitations to consider, including the inability to comprehensively identify StUD based on administrative health data. ICD 9 or ICD-10-CA codes in MSP and DAD were used to identify StUD. In the ICD 9, four-digit codes for drug dependence or non-dependent use are required to identify the substance(s) the person uses. However, many records in MSP only have 3-digit ICD 9 codes likely due to data quality issues or provider practice.

Thus, we are likely missing cases due to lack of a 4^th^ digit specifying stimulant use. It is unknown whether these data quality issues would have a differential impact on records post-pandemic compared to pre- pandemic. Some of these challenges with other substances such as opioids can be alleviated due to medication records for opioid agonist treatment (OAT) that can be used in case definitions. However, medication treatment for StUD is not widely available, additionally these medications are difficult to distinguish in pharmacy databases from stimulants prescribed for other reasons (e.g. ADHD, narcolepsy).

It is important to acknowledge that the identified trends may be impacted by a potential reduction in health and substance use services during the pandemic, that could artificially deflate counts during the pandemic, and create an artificial inflation post-pandemic. This is a natural limitation of using administrative health data, which relies on contact with health services to identify incident diagnoses.

It’s likely that the diagnoses from MSP and DAD will represent the most severe cases of StUD. People with mild to moderate StuD may not be identified by these codes. As well cases with prominent psychosis related features may be misdiagnosed.

There is insufficient detail in ICD codes to distinguish between prescription and unregulated StUD. Furthermore rates of ADHD diagnosis in BC and Canada are increasing (54). Given the known co-occurrence of ADHD and StUD there is a possibility of missed, or underdiagnosed StUD among people with ADHD. It is likely that diagnoses from administrative health datasets used in this study will represent the most severe cases of StUD. People with mild to moderate StuD may not be identified by these diagnostic codes. It is also possible that people with prominent psychosis related features may be misdiagnosed.

## Conclusion

We identified a rate of increase in incident StUD diagnoses since the COVID-19 pandemic declaration in BC that was not statistically significant; however, there is still a small gradual increase and the rate of incident StUD has been increasing overall since before the pandemic. These findings underscore the importance of considering service planning, support and access to treatment, for people with StUD in particular with people who have co-occurring ADHD, cardiac conditions and/or kidney disease, as the proportion of cases with physician visits for these conditions were higher in the post pandemic period. Increased efforts are needed to understand trends of stimulant use at a population-level. Our study highlights the need for more comprehensive linked data -including, administrative health data, surveys, and other services/program data (e.g., community services, private sector) to better disentangle StUD incidence and prevalence to inform services to meet the needs of people with StUD.

## Data Availability

The raw data supporting the conclusions of this article will be made available by the authors, without undue reservation.
