# Supplementary material for: Trends of incident stimulant use disorder diagnoses before and after the COVID-19 pandemic in British Columbia (2013-2024): a population-based study
Source: Front Psychiatry. 2026 May 7;17:1682481. doi: 10.3389/fpsyt.2026.1682481 (PMC13190574; doi:10.3389/fpsyt.2026.1682481)
Supplement: Supplementary file 1 [file SupplementaryFile1.docx]

**Table S1**. ICD-10-CA and ICD-9 Codes for Identification of Stimulant Use Disorder

A person was defined as a StuD case if they had two outpatient records within a year, in MSP or 1 hospitalization record in DAD with ICD-9 codes (for MSP) or ICD-10-CA codes (DAD) as listed in the table below.

|  | **Hospitalizations,**  **(ICD-10-CA)** | **Outpatient care (ICD-9)** |
| --- | --- | --- |
| Mental and Behavioural disorders due to use of cocaine  Mental and Behavioural disorders due to use of other stimulants including caffeine | F14 (0-9)  F15 (0-9) |  |
| Drug Dependance: Cocaine  Drug Dependance: Amphetamine type and other Psychostimulants  Non-dependent [use] of drugs: Cocaine Type  Non-dependent [use] of drugs: Amphetamine Type |  | 304.2  304.4  305.6  305.7 |

**Table S2**. ICD-9 Codes for Identification of Physician Visits for Chronic Illness, Mental Health Visits, Substance Use visits. Extracted From BCCDC Chronic Disease Dashboard:
<http://www.bccdc.ca/health-professionals/data-reports/chronic-disease-dashboard#Case--definitions>

| **Reason for Visit** | **ICD 9 Code Description** | **ICD-9 Code** | **Other Codes** |
| --- | --- | --- | --- |
| **ADHD** | Hyperkinetic syndrome of childhood | 314 (0-9) |  |
| **Depression** | Affective Psychoses Depressive Disorder, Not elsewhere classified Anxiety/Depression | 296 (0-9) 311  50B |  |
| **Mood & Anxiety** | Affective Psychoses Neurotic Disorders Depressive Disorder, Not elsewhere classified Anxiety/Depression | 296 (0-9) 300 (0-9) 311  50B |  |
| **Opioid Use Disorder** | Drug Dependence: Morphine Type Drug Dependence: Combinations of Morphine Type with Any Other Nondependent [use] of drugs: Morphine Type | 304.0 304.7   305.5 | MSP Fee items:  39, 15039, 13013, 13014, 36521 |
| **Substance Use Disorder (excluding stimulant use disorder)** | Drug Dependence  Nondependent drug [use] | 304 (0, 1, 3, 5, 6, 7, 8, 9)  305 (0-5, 8, 9) |  |
| **Schizophrenia** | Schizophrenic Psychoses | 295 (0-9) |  |
| **Osteoarthritis** | Osteroarthritis and Allied Disorders | 715 |  |
| **Rhematoid Arthritis** | Rheumatoid Arthritis And Other Inflammatory Polyarthropathies | 714 |  |
| **Asthma** | Asthma | 493 (0-9) |  |
| **COPD** | Chronic Bronchitis Emphysema Chronic Airways Obstruction. Not elsewhere Classified | 491 (0, 1, 9) 492   496 |  |
| **Diabetes** | Diabetes Mellitus | 250 (0-9) |  |
| **Heart Disease** | Acute Myocardial Infarction Other Acute And Subacute Forms Of Ischemic Heart Disease Old Myocardial Infarction  Angina Pectoris  Other Forms Of Chronic Ischaemic Heart Disease (0-9) | 410  411  412 413  414 |  |
| **Heart Failure** | Heart Failure | 428 |  |
| **Hypertension** | Essential Hypertension Hypertensive Heart Disease Hypertensive Renal Disease Hypertensive Heart and Renal Disease Secondary Hypertension | 401 402 403 404 405 |  |
| **Kidney Disease** | Hypertensive Renal Disease Nephrotic Syndrome Chronic Glomerulonephritis Nephritis and Nephropathy Acute Renal Failure Chronic Renal Failure Renal Failure, Unspecified Renal Sclerosis, Unspecified Diabetes With Renal Manifestations Hypertensive Heart and Renal Disease Congenital Anomalies of Kidney, Ureter Urethra & Bladder Neck, Urachus Kidney Diseases Persons with Conditions Influencing their health status – Kidney  Aftercare Involving Intermittent Dialysis Aftercare Kidney Persons without reported diagnosis encountered during examination – Nephropathy, Other & Unspecified Genitourinary conditions  -- --  Persons with Potential Health Hazards Related to Personal And Family History: Disorders of the Urinary System Persons with Conditions Influencing their health status – Renal Dialysis Status  -- | 403 (0-9) 581 (0-9)  582 (0-9)  583 (0-9)  584 (0-9)  585 (0-9)  586 (0-9)  587 (0-9) 250.4  404(0-9)  753 (2, 4, 6-9) V186  V420  V56 (0, 8) V59.4  V81 (5, 6)  2494 (0, 1)  28521  V130 (3-9)  V451 (0-2)  V4573 |  |

**Table S3.** ICD-10-CA Codes for Identification of Physician Visits for Chronic Illness, Mental Health Visits, Substance Use visits. Extracted From BCCDC Chronic Disease Dashboard: <http://www.bccdc.ca/health-professionals/data-reports/chronic-disease-dashboard#Case--definitions>

| **Reason for Visit** | **ICD 10-CA Code Description** | **ICD-10-CA Code** | **Other Codes** |
| --- | --- | --- | --- |
| **ADHD** | Hyperkinetic Disorders | F90 |  |
| **Depression** | Depressive Episode  Recurrent Depressive Disorder | F32 F33 |  |
| **Mood & Anxiety** | Manic Episode Bipolar Affective Disorder Depressive Episode  Recurrent Depressive Disorder Persistent Mood Disorders Other Mood Disorders Unspecified Mood Disorder Phobic Anxiety Disorders  Other Anxiety Disorders  Obsessive-compulsive Disorder  Reaction to severe stress and adjustment disorders  Dissociative Disorders Somatoform Disorders  Other Neurotic Disorders Other Disorders Of Adult Personality And Behaviour | F30  F31  F32  F33  F34  F38  F39  F40  F41  F42  F43  F44  F45  F48  F68 |  |
| **Opioid Use Disorder** | Mental and Behavioural disorders due to use of opioids | F11 |  |
| **Substance Use Disorder (excluding stimulant use disorder)** | Mental and Behavioural disorders due to psychoactive substance use | F10-F19 |  |
| **Schizophrenia** | Schizophrenia Schizotypal Disorder Acute and Transient Psychotic Disorders  Schizoaffective Disorders | F20  F21  F23  F25 |  |
| **Osteoarthritis** | Arthrosis | M15-M19 |  |
| **Asthma** | Asthma | J45 |  |
| **COPD** | Simple and Micopurulent Chronic Bronchitis  Unspecified Chronic Bronchitis Emphysema  Other Chronic Obstructive Pulmonary Disease | J41  J42  J43  J44 |  |
| **Diabetes** | Diabetes Mellitus | E10-E14 |  |
| **Heart Disease** | Ischemic Heart Diseases | I20-I25 |  |
| **Heart Failure** | Heart Failure | I50 |  |
| **Hypertension** | Hypertensive Diseases | I10-I15 |  |
| **Kidney Disease** | Rapidly Progressive Nephritic Syndrome  Chronic Nephritic Syndrome  Nephrotic Syndrome  Unspecified Nephritic Syndrome  Isolated Proteinuria with Specified Morphological Lesion  Hereditary Nephropathy, not elsewhere classified  Chronic Kidney Disease  Unspecified Kidney Failure  Unspecified Contracted Kidney  Small Kidney of Unknown Cause | N01  N03  N04  N05  N06  N07  N18  N19  N26  N27 |  |

**Table S4: Data Source Citations**

| British Columbia Ministry of Health [creator] (2025). Client Roster (Client Registry System/Enterprise Master Patient Index). British Columbia Ministry of Health [publisher]. Data Extract. Ministry of Health (2021). <https://www2.gov.bc.ca/gov/content/health/health-forms/online-services>  Canadian Institute of Health Information [creator] (2025): Discharge Abstract Database (Hospital Separations). British Columbia Ministry of Health [publisher]. Data Extract. MOH (2020). <http://www2.gov.bc.ca/gov/content/health/conducting-health-researchevaluation/data-access-health-data-central>  British Columbia Ministry of Health [creator] (2025). Medical Services Plan (MSP) Payment Information File. British Columbia Ministry of Health [publisher]. Data Extract. Ministry of Health (2021). <https://www2.gov.bc.ca/gov/content/health/health-forms/online-services>  British Columbia Ministry of Health [creator] (2025). PharmaNet. British Columbia Ministry of Health [publisher]. Data Extract. Ministry of Health (2021). <https://www2.gov.bc.ca/gov/content/health/health-forms/online-services>  British Columbia Ministry of Citizen Services. Population estimates - province of british columbia [Internet]. British Columbia Ministry of Citizen Services; 2025 [cited 2025 Jul 18]. Available from: https://www2.gov.bc.ca/gov/content/data/statistics/people-population-community/population/population-estimates |
| --- |

**Table S1:** Plot of the residuals of Arima for StUD data from January 1, 2013 – February 28, 2020


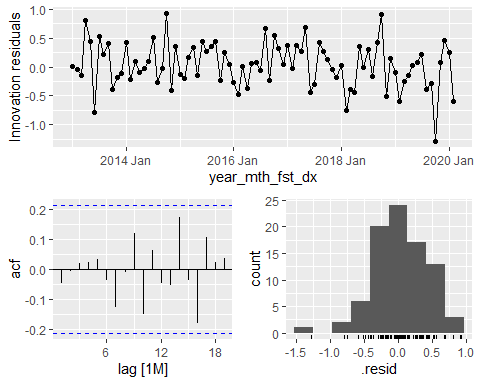


**Figure S2**: New StuD Diagnoses by Sex and month in BC (January 1, 2013- December 31, 2024)


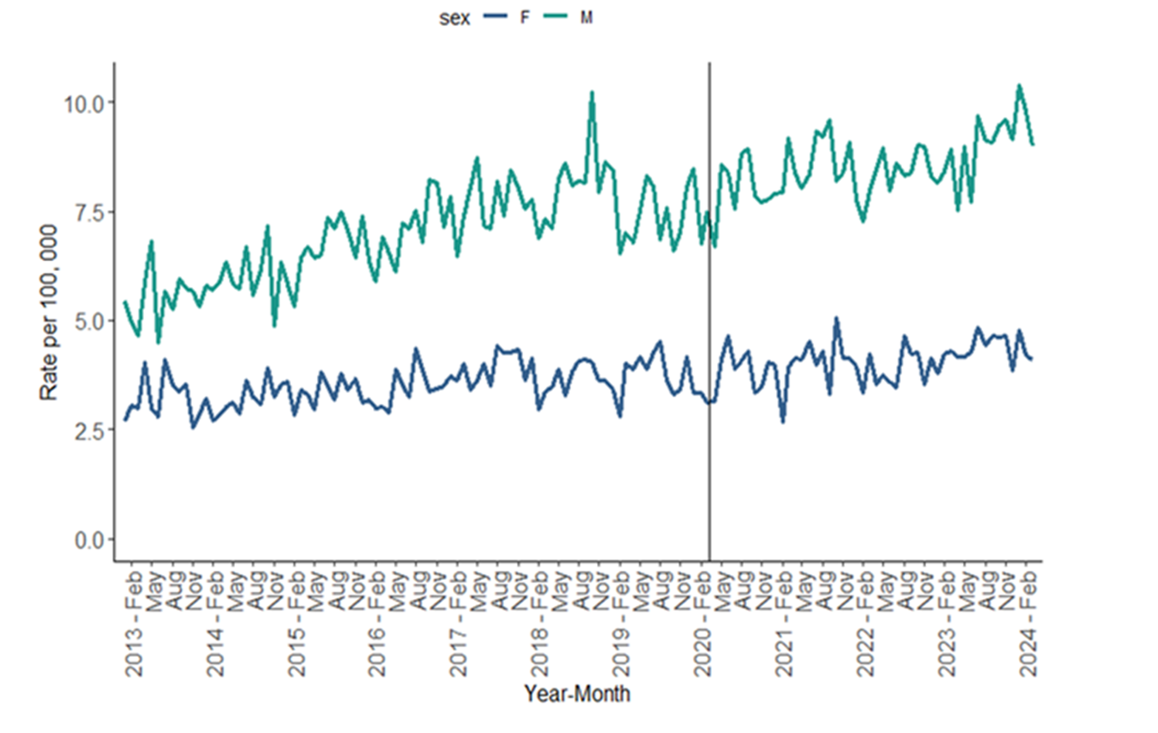


Footnote: F= Female; M= Male
